# Molt-inhibiting hormone stimulates vitellogenesis at advanced ovarian developmental stages in the female blue crab, *Callinectes sapidus *1: an ovarian stage dependent involvement

**DOI:** 10.1186/1746-1448-5-7

**Published:** 2009-07-07

**Authors:** Nilli Zmora, John Trant, Yonathan Zohar, J Sook Chung

**Affiliations:** 1 Center of Marine Biotechnology, University of Maryland Biotechnology Institute, 701 E. Pratt St. Columbus Center Suite 236, Baltimore, MD 21202, USA

## Abstract

To understand the hormonal coordination of the antagonism between molting and reproduction in crustaceans, the terminally anecdysial mature female *Callinectes sapidus *was used as a model. The regulatory roles of crustacean hyperglycemic hormone (CHH) and molt-inhibiting hormone (MIH) in vitellogenesis were examined. A competitive specific RIA was used to measure the levels of MIH and CHH in the hemolymphs of mature females at pre- and mid- vitellogenic stages, and their effects on vitellogenesis at early (early 2, E2) and mid vitellogenesis (3) stages were determined *in vitro*. A hepatopancreas fragments incubation system was developed and the levels of vitellogenin (VtG), as well as *VtG *mRNA and *heterogeneous nuclear (hn)VtG *RNA were determined using RIA or QPCR, respectively. MIH titers were four times higher at mid-vitellogenesis than at pre-vitellogenesis, while CHH levels in the hemolymph were constant. In the *in vitro *incubation experiments, MIH increased both *VtG *mRNA levels and secretion at ovarian stage 3. At stage E2, however, MIH resulted in a mixed response: downregulation of *VtG *mRNA and upregulation of *hnVtG *RNA. CHH had no effect on any of the parameters. Actinomycin D blocked the stimulatory effects of MIH in stage 3 animals on *VtG *mRNA and VtG, while cycloheximide attenuated only VtG levels, confirming the MIH stimulatory effect at this stage. MIH is a key endocrine regulator in the coordination of molting and reproduction in the mature female *C. sapidus*, which simultaneously inhibits molt and stimulates vitellogenesis.

## Background

It has been proposed in decapod crustaceans that the high energy demanding processes of molting and reproduction are mutually antagonistic and do not occur simultaneously [1]. The antagonism is clearly demonstrated in the female *Callinectes sapidus*: molting and reproduction occur in two distinctive life phases with molting cycles occurring only in the juvenile phase and ovarian development and spawning cycles occurring only in adulthood.

Ovarian development is a major reproductive process, during which oocytes grow as a result of vitellogenin production and its accumulation in the form of yolk protein (vitellin) and other cytoplasmic egg proteins in the oocytes [2]. In *C. sapidus*, vitellogenesis occurs primarily in the hepatopancreas after terminal/pubertal molt and requires a relatively short duration of 8–12 weeks for completion [3].

The phenomenon of reproductive phase accompanied by terminal anecdysis (halt of molting cycles) is found in only a few decapod crustaceans, including *Maja squinado, Libinia emarginata*, and *Chionoecetes bairdi *[4-7]. Most of these anecdysial animals are characterized by a degenerated molting gland, the Y-organ (YO), and the resulting low levels of molting hormones (ecdysteroids) in the hemolymph [8]. However, the adult *C. sapidus *female is distinguished from these animals by having non-degenerated YO's, which retain their normal size and their capability to bind and respond to molt-inhibiting hormone (MIH) by elevation of cyclic GMP (cGMP). Furthermore, eyestalk ablation (the removal of the source for MIH) induces molting in adult *C. sapidus *females [9], indicating that the YO's also preserve their steroidogenic potential. Taken together, unlike other anecdysial crustacean species, molting cycle in the mature female *C. sapidus *is temporarily arrested at intermolt stage, in synchrony with the reproductive phase. Since to date the neuroendocrine mechanism underlying these antagonistic processes has not been examined in any crustacean species, the mature *C. sapidus *female with its distinctive separate molting and reproductive phases, serves as an excellent model species to address the issue.

Molting and reproduction are regulated by the eyestalk derived crustacean hyperglycemic hormone (CHH) family [10,11] including CHH, MIH, mandibular organ-inhibiting hormone (MOIH) [12], and gonad/vitellogenesis-inhibiting hormone (G/VIH) [13,14]. These neuropeptides are synthesized in specific neurosecretory cells of the medullar terminalis X-organ (XO), stored, and released from the sinus gland (SG) in the eyestalk ganglia (XOSG complex). The names of these neuropeptides usually refer to their primary functions or structural affiliation to either CHH or MIH subfamilies.

CHH and MIH exist in different combinations and number of isoforms depending on the species. Multiple isoforms are found in the XOSG of penaeids, crayfish, and lobsters [15], whereas in crab species only two isoforms of CHH and one of MIH are detected [16-19]. Moreover, CHH neuropeptides are characterized by pleiotropicity and are usually involved in the regulation of several physiological processes including molting, reproduction, osmoregulation, energy metabolism, and stress response [20].

Molting is controlled by CHH and MIH. High circulating levels of these neuropeptides suppress synthesis and secretion of ecdysteroids from the YO's by down-regulating protein synthesis [21,22] which in turn leads to low titers of ecdysteroids in the hemolymph [7,23-25]. Reproduction on the other hand is regulated both negatively and positively by CHH family neuropeptides. The inhibition is exerted by VIH in the eyestalk ganglia [14,26,27], and its presence has been described in a few lobster species [28-31], *Litopenaeus vannamei *[32], and the isopod *Armadillidium vulgare *[33]. Vitellogenesis-stimulating hormone (VSH), identified as an isoform of MIH in the sand shrimp, *Metapenaeus ensis *[34], positively regulates vitellogenesis. In addition to the XOSG, it is expressed in the ventral nerve cord, thoracic ganglia, and brain. This finding implies that a MIH like neuropeptide may be involved in the regulation of reproduction.

Considering the roles of CHH and MIH in molting and MIH isoform action as a VSH, we aimed to understand how the antagonism between molting and reproduction is hormonally coordinated in the terminally anecdysial female *C. sapidus*. Our earlier study showed that like other crab species, only one form of MIH is identified in the XOSG of *C. sapidus *[35] and *MIH *mRNA [36]. In addition, we found no evidence of a MIH, MIH-like neuropeptide(s) or its mRNA in the thoracic ganglia or brain of the adult female (unpublished data). We therefore hypothesized that XOSG derived CHH or MIH actively inhibit molting in the female *C. sapidus*, while allowing reproductive processes to take place. Specifically, we questioned if MIH or CHH have a role in the regulation of vitellogenesis of *C. sapidus*. To address these questions, we first determined the hemolymph circulating concentrations of MIH and CHH and their mRNA levels in the XO of mature female *C. sapidus*. We also carried out *in vitro *studies on hepatopancreas tissue fragments to identify the regulatory roles of MIH and CHH in vitellogenesis, specifically at the levels of *VtG *mRNA, VtG protein and *heterogeneous nuclear (hn) VtG *RNA. Measuring *hnVtG *RNA provided an additional tool to examine transcription. We report that MIH has a regulatory role in vitellogenesis in the female *C. sapidus*, while CHH seems to have no clear involvement. Specifically, MIH upregulated *VtG *mRNA, *hnVtG *RNA and VtG secretion in heptopancreas fragments *in vitro *in the advanced vitellogenic stage. Interestingly, MIH downregulated *VtG *mRNA, upregulated *hnVtG *RNA and had no effect on VtG secretion in the early vitellogenic stage.

## Results

### MIH and CHH titers in the hemolymph of reproductive females

**Figure 1:**
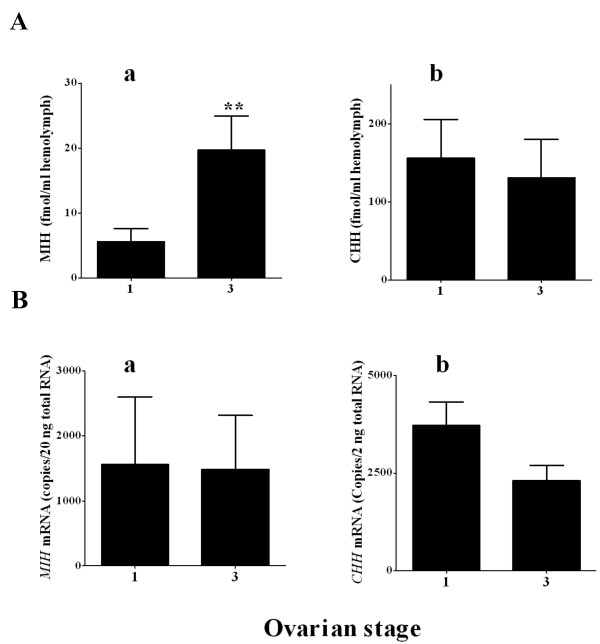
**MIH and CHH levels in the hemolymph and mRNAs in the XO of pre- and mid-vitellogenic females (ovarian stage 1 and 3)**. A) hemolymph titers and B) mRNA a) MIH, b) CHH. MIH and CHH neuropeptides in the hemolymph were extracted with 40% isopropanol for the extraction of CHH and 60% isopropanol for the extraction of MIH followed by a specific RIA in 10 females at each stage 1 or 3. *MIH *and *CHH *mRNA levels were determined using QPCR in 17 females at stage 1 and 21 females at stage 3 and are presented as copies/20 ng total RNA. Results are presented as mean ± SEM (N = 10). **, = P ≤ 0.01.

Neuropeptides in the hemolymph were determined by specific radioimmunoassay (RIA) in 10 females at ovarian stages 1 and 3, representing pre-vitellogenic and mid-vitellogenic stages, respectively. The results showed a significant four- fold increase in MIH levels at ovarian stage 3 (19.6 fmol/ml) compared to stage 1 (5.7 fmol/ml) (Fig. 1Aa). CHH levels did not differ between the stages and ranged between 130 and 150 ± 49 fmol/ml which are ~6 – 20 times higher than those of MIH (Fig. 1Ab). *MIH *and *CHH *mRNA levels in the XO of mature females at the same stages did not significantly differ and were 1500–2500 copies/20 ng total RNA for MIH and 15000–40000 copies/20 ng total RNA for CHH (Figs. 1Ba and 1Bb). Accordingly, the concentrations of ecdysteroids in the hemolymph of these females were low at ovarian stages 1 and 3: 7 to 5 ng/ml, respectively (not shown).

### *In vitro *incubation of hepatopancreas fragments

#### The effects of sinus gland neuropeptides (CHH and MIH) on *VtG *expression and secretion

**Figure 2:**
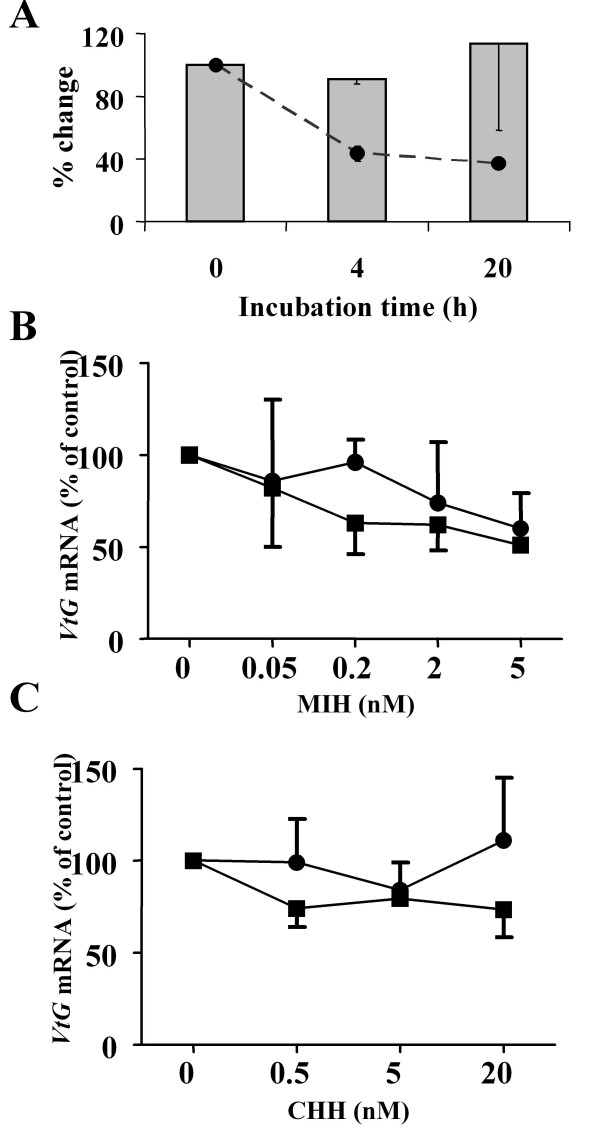
**Evaluation and optimization of the hepatopancreas incubation experiment**. A) Incubation period had no effect on the stability of *VtG *mRNA in hepatopancreas fragments of females at early ovarian stage 2 (E2). Bars, normalized *VtG *mRNA; dashed line, total RNA. MIH had a more pronounced decreasing effect on *VtG *mRNA levels than CHH (0 – 20 nM) in hepatopancreas fragments of females at E2 incubated for 1 and 6 h, B) Incubation with 0 to 5 nM MIH and C) Incubation with 0 to 20 nM CHH. circles, 1 h incubation; squares, 6 h incubation. Data are presented as mean ± SEM (N = 3).

To establish an *in vitro *bioassay, a preliminary experiment was conducted to determine the effect of incubation period on the stability of total RNA and *VtG *mRNA as well as the optimal incubation time and dose of neuropeptides for hepatopancreas fragments *in vitro*. The stability of RNA was tested by visualization of the total RNA, determined by the integrity of 18 S and 28 S ribosomal(r) RNA subunits of the hepatopancreas extracted immediately after dissection (t = 0 point) and after a 6 h incubation. The results presented in additional file 1 show that 18 S and 28 S rRNA bands remained stable and similar to that of t = 0 point after the 6 h incubation. The levels of *VtG *mRNA were further tested by quantitative PCR (QPCR) analysis on hepatopancreas fragments incubated in plain media. Levels were found to be constant during the incubation period (Fig. 2A).

The effect of different doses of CHH or MIH on *VtG *mRNA was determined by incubating hepatopancreas fragments of stage (early 2) E2 animals for 1 or 6 h. At 1 h incubation, MIH at 0.05, 0.2 and 2 nM concentrations had no effect, however 5 nM resulted in a non-statistically significant decrease of 25% of control in *VtG *mRNA. During the 6 h incubation, the effect was more pronounced, although still statistically insignificant due to the low N number (N = 3). MIH at 0.2, 2, and 5 nM showed a trend of dose dependent effect, where *VtG *mRNA levels dropped to 62%, 62%, and 51% of the control, respectively (Fig. 2B). CHH at 0.5, 5, and 20 nM showed no difference from control on *VtG *mRNA levels at both 1 and 6 h incubation (Fig. 2C). Based on these results, the subsequent *in vitro *experiments were carried out at 6 h incubation, unless specified otherwise.

**Figure 3:**
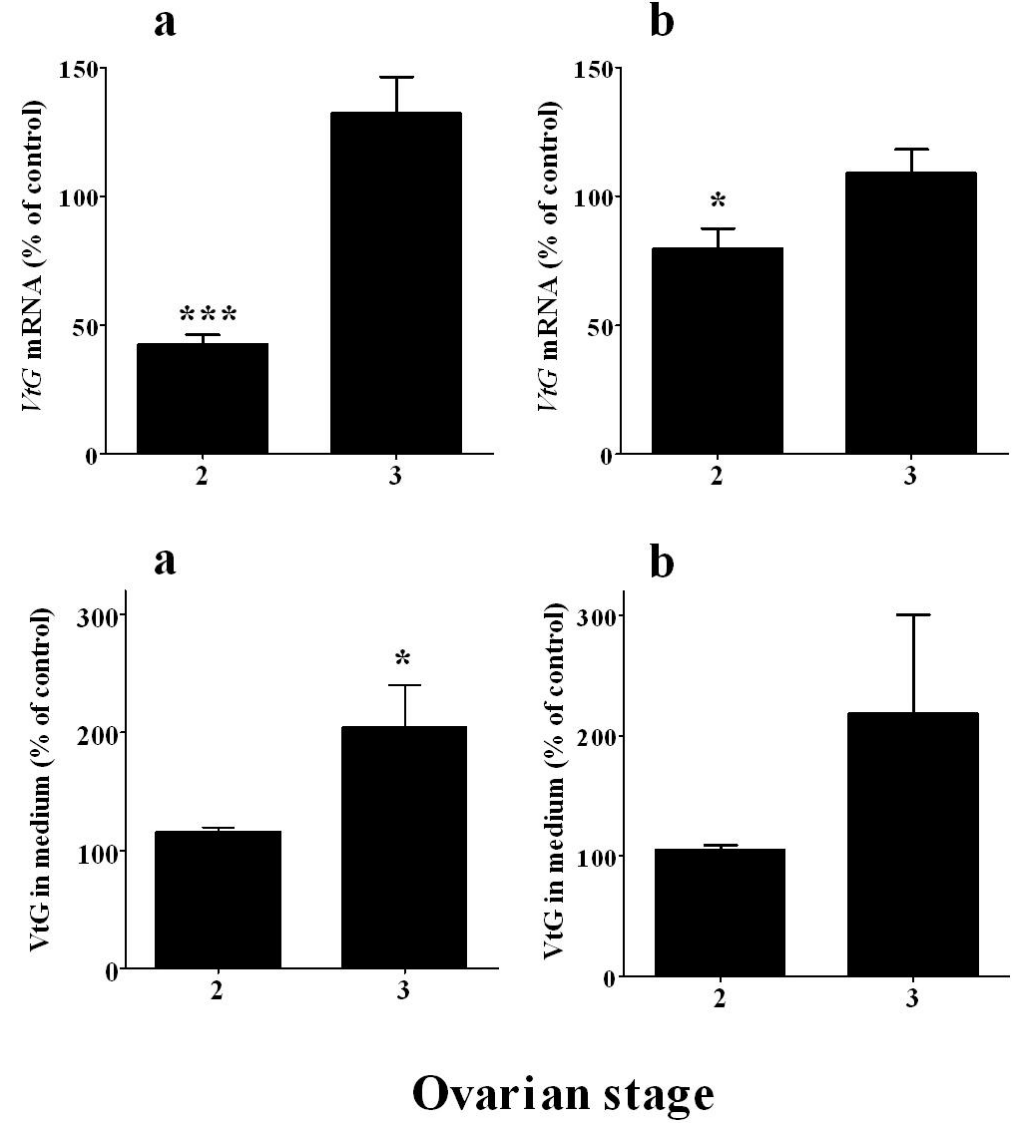
**MIH stimulates *VtG *expression and secretion in hepatopancreas of females at ovarian stage 3 and downregulates *VtG *mRNA at stage early 2 (E2) while CHH shows no significant effect**. A) *VtG *mRNA and B) VtG in the medium. a) 2 nM MIH treatment and b) 20 nM CHH treatment. The effects on *VtG *mRNA and protein secretion were tested in females at E2 (N = 21 and N = 6, respectively) and stage 3 (N = 6 and N = 5, respectively). Results are presented as mean ± SEM of % of control. * = P ≤ 0.05; *** = P ≤ 0.001.

The effects of CHH and MIH on vitellogenesis were further examined on hepatopancreas of females at early and mid-vitellogenic stages (E2 and 3, respectively). CHH (20 nM) significantly reduced *VtG *mRNA levels by 20% (N = 21) of control at E2 and had no effect at stage 3 (N = 6) (Fig. 3Ab). MIH however, revealed a stage dependent response. MIH (2 nM) reduced *VtG *mRNA levels in the hepatopancreas of females at E2 by 60% (N = 21), but caused an increase at stage 3 (N = 6) (Fig. 3Aa). The effect of MIH on VtG secretion from hepatopancreas fragments was stage dependent: at E2, no change was detected (N = 6), while at stage 3, MIH increased the levels of VtG in the media to 204 ± 34% over the control (N = 5) (Fig. 3Ba). CHH had no significant effect on VtG secretion at E2 and 3 stages (N = 5)(Fig. 3Bb).

**Figure 4:**
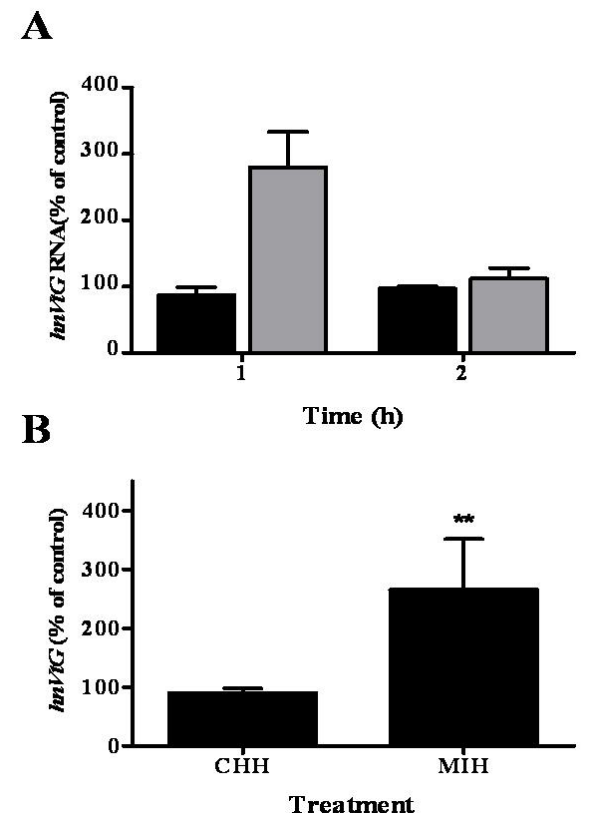
**MIH upregulates *hnVtG *RNA in hepatopancreas of females at ovarian stage E2 after 1 h incubation**. A) Changes in *hnVtG *RNA were measured after 1 h or 2 h incubation with either 20 nM CHH (black bars) or 2 nM MIH (grey bars) and are measurable only at 1 h incubation period (N = 3). B) *hnVtG *RNA change in response to 20 nM CHH or 2 nM MIH (N = 11). Results are presented as mean ± SEM of % of control. * = P ≤ 0.05; ** = P ≤ 0.01.

To clarify the results obtained for stage E2, the effect of CHH and MIH on VtG transcription was also tested on *de novo *synthesis of *VtG *mRNA by measuring heterogeneous nuclear RNA of *VtG *(*hnVtG *RNA). The levels of *hnVtG *RNA increased to 270% over the control with MIH, whereas CHH had no effect after a 1 h incubation. This effect disappeared at the 2 h incubation period (N = 3) (Fig. 4A). Since a difference in *hnVtG *RNA was only measurable at the 1 h but not 2 h incubation, experiments testing *hnVtG *RNA were set for 1 h. In a larger scale set of experiments with a 1 h incubation, MIH caused a ~2.5 fold increase (256 ± 86%) in *hnVtG *RNA levels in the hepatopancreas of females at E2, while CHH had no effect (N = 11) (Fig. 4B).

#### The effects of co-incubation of actinomycin D and cycloheximide with MIH on *VtG *expression and VtG production

**Figure 5:**
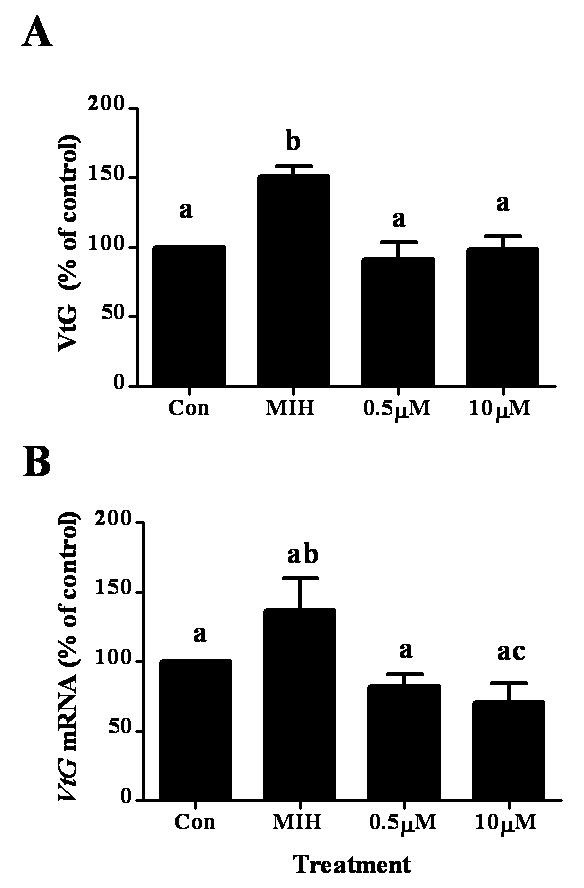
**Co-incubation of MIH and actinomycin D (AD) blocks the increase of VtG protein (A) and *VtG *mRNA (B) in hepatopancreas fragments of ovarian stage 3 females**. The concentration of MIH was 2 nM and those of actinomycin D were 0.5 and 10 μM. Results are presented as mean ± SEM of % of control (N = 5). The alphabetical letters show the significant differences at P < 0.05.

To test whether the changes in VtG secretion by MIH is associated with its impact on *VtG *mRNA, hepatopancreas fragments of females at ovarian stage 3 were co-incubated with 2 nM MIH and a transcription inhibitor (actinomycin D) or a translation inhibitor (cycloheximide). These inhibitors were tested at two different concentrations: 0.5 and 10 μM. MIH increased VtG levels by 51% compared to the control and MIH + 0.1% v/v EtOH increased by 45% (N = 5). Actinomycin D at 0.5 and 10 μM, in the presence of MIH, reduced VtG levels to 60% and 49% of MIH treatment, respectively (Fig. 5A), which were similar to the media control. MIH + 10 μM AD decreased *VtG *mRNA to 51% of MIH treatment (Fig. 5B).

**Figure 6:**
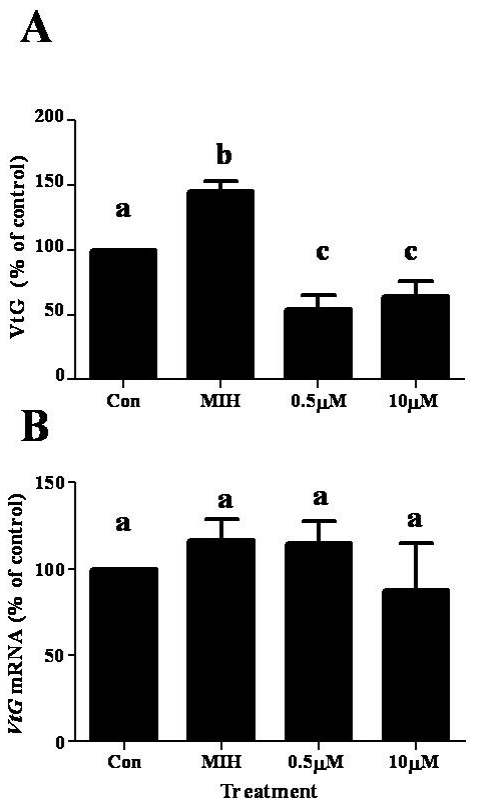
**Co-incubation of MIH and cycloheximide reversed the effect of MIH on VtG protein (A) and had no effect on *VtG *mRNA (B)**. The concentration of MIH was 2 nM and those of cycloheximide were 0.5 and 10 μM. Results are presented as mean ± SEM of % of control (N = 5). The alphabetical letters show the significant differences at P < 0.05.

Cycloheximide at 0.5 and 10 μM co-incubated with MIH (+ EtOH), reduced VtG levels to ~35% of MIH (+ EtOH) and ~54% of the media control, but had no effect on *VtG *mRNA levels (Fig. 6A and 6B). *HnVtG *RNA was not measured in this particular set of experiments as a different experimental setup is required (i.e., 1 h incubation time, see Fig. 4A).

#### The effect of other sinus gland factors on *VtG *mRNA levels

**Figure 7:**
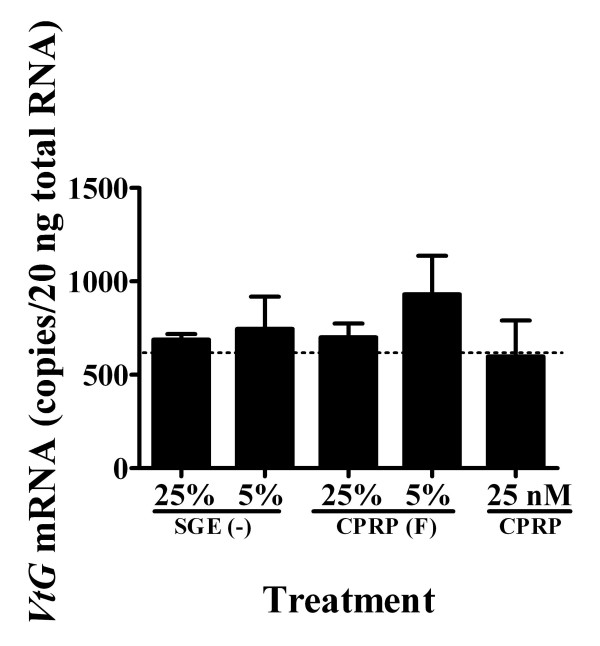
**Sinus gland extract (SGE) without the known neuropeptides caused no change in *VtG *mRNA in hepatopancreas of stage 3 *in vitro***. Hepatopancreas fragments were incubated with sinus gland extract from which CPRP, CHH, and MIH were removed (SGE(-)) at 25% and 5% sinus gland equivalent; CPRP HPLC fraction (CPRP F) at 25% and 5% sinus gland equivalent; and at 25 nM CPRP. Control level is indicated by a dashed line and data are presented as mean ± SEM (N = 5).

The pooled HPLC fractions of sinus-glands (SG) from stage 3 vitellogenic females without CHH, MIH, and crustacean hyperglycemic hormone precursor related- peptide (CPRP) were tested using an *in vitro *hepatopancreas incubation to determine whether other sinus gland-derived substances had an effect on *VtG *expression. No effect was found with 5% and 25% of SG equivalent (minus CHH, MIH, and CPRP) on *VtG *expression in the hepatopancreas fragments (Fig. 7). CPRP was also tested, as it is one of the three major neuropeptides in the SG. CPRP fraction at 5% and 25% SG equivalents and purified native CPRP at 25 nM showed no significant effect on *VtG *expression (Fig. 7).

## Discussion

The present study aimed to gain a better understanding of how XOSG derived neuropeptides regulate the antagonism between vitellogenesis and molting in the terminally anecdysial mature female *C. sapidus*. We determined the titers of CHH and MIH in the hemolymph of mature females at different vitellogenic stages and tested their effect on vitellogenesis, *in vitro*. Hemolymph titers of CHH in these females were comparable to those measured in juvenile animals [35] and remained unchanged at ovarian stages 1 and 3. However, the detection of MIH in the hemolymph of mature females was initially surprising considering they ceased molt. Hemolymph MIH levels at mid-vitellogenic ovarian stage 3 were four times higher than those of stage 1 (Fig. 1A), implying a putative role for MIH in vitellogenesis. *MIH *and *CHH *mRNA levels were statistically indifferent in both ovarian stages, with MIH exhibiting high variation within each group (Fig. 1B). These results indicate that the expression level of these neuropeptides is not correlated with their secretion from the SG, as shown in other studies [22,36].

The *in vitro *hepatopancreas fragments incubation system was used to further determine whether circulating CHH and MIH play a regulatory role in vitellogenesis in *C. sapidus *[5,27,28,32,34,37,38]. Their effect was tested at the levels of VtG gene transcription by measuring changes in *VtG *mRNA levels, and VtG translation, determined by changes in VtG secretion as this protein does not accumulate in the hepatopancreas but is being secreted immediately following translation [3]. CHH had no consistent effects on vitellogenesis: it decreased *VtG *mRNA by 20% only at stage E2 and had no effect on VtG secretion at both E2 and 3 stages (Figs. 3Ab and 3Bb). However, with MIH treatment, both *VtG *mRNA and VtG protein levels exhibited different responses at the two ovarian stages tested. While *VtG *mRNA decreased at stage E2, it slightly increased at stage 3 in response to MIH (Fig. 3Aa). VtG secretion did not differ from control treatment at stage E2 but increased at stage 3 (Fig. 3Ba). These results imply that MIH may have an inhibitory role at early ovarian stages, and a stimulatory role at mid ovarian stages. To determine whether MIH indeed downregulates *VtG *gene transcription at stage E2, we further tested the activation of *VtG *gene by measuring the short-lived *hnVtG *(= pre *VtG *mRNA). The increase of *hnVtG *RNA levels in response to MIH at this stage indicated that the *VtG *gene is activated and thus its transcription is being enhanced (Fig. 4). These results were corroborated by a different set of experiments in which incubation of hepatopancreas fragments with membrane permeable cyclic nucleotides at stage E2 resulted in the same response [39]. The reason for the contradicting response of *VtG mRNA *and *hnVtG *RNA is unclear and will require further study.

Stimulation of vitellogenesis by SG-MIH has not been reported in decapod crustaceans, with the exception of MIH-B in *M. ensis *[34]. To verify the stimulatory effect of MIH on vitellogenesis at ovarian stage 3, actinomycin D (a general inhibitor of eukaryotic transcription) and cycloheximide (a general inhibitor of eukaryotic translation), were separately co-incubated with MIH. The addition of actinomycin D and cycloheximide to MIH treatment resulted in a decrease to 60% and 35% of VtG levels, respectively, compared to MIH alone (equivalent to 95% and 54% of control levels) (Figs. 5 and 6). Actinomycin D co-incubated with MIH, decreased *VtG *mRNA levels by 49%, indicating that the respective decrease in VtG levels is likely to be a result of the reduced *VtG *transcription (Fig. 5A). The decrease of *VtG *mRNA with MIH/actinomycin D to control levels suggests pre-existing *VtG *mRNA in the hepatopancreas fragments. Furthermore, cycloheximide probably blocked the translation of the newly MIH induced *VtG *mRNA as well as the pre-existing *VtG *mRNA, resulting in lower levels of VtG protein compared to the control. In addition, the unchanged levels of *VtG *mRNA with MIH/cycloheximide imply that the transcription machinery was not affected. Overall, these results demonstrate that MIH stimulation occurs at transcription and translation of VtG at ovarian stage 3.

Support evidence for MIH regulation in vitellogenesis is provided by the appearance of specific binding sites for MIH in the hepatopancreas of mature female *C. sapidus*. The abundance of the binding site increased gradually with ovarian development and was higher at stage 3 than at stages 1 and 2 [39].

It is tacitly accepted that V/GIH inhibits vitellogenesis, while vitellogenin/gonad-stimulating hormone (V/GSH) enhances vitellogenesis processes. However, CHHs were shown to inhibit general mRNA synthesis in the ovaries of vitellogenic female *Penaeus semisulcatus *[40]. The few cases reporting the decrease of *VtG *mRNA levels by V/GIH and CHH have been shown in ovaries of immature penaeid females [28,32,41]. Although these studies were conducted on ovarian tissue and not hepatopancreas, they support our finding of decreased *VtG *mRNA levels and indicate that this effect may be more common at early stages of ovarian development.

The observation that MIH positively regulates vitellogenesis at stage 3 is concordant with a previous report in which the non SG-MIH isoform stimulated vitellogenesis in the shrimp *M. ensis *[34]. The lack of MIH or MIH-like neuropeptide in non-XOSG tissues of the female *C. sapidus*, suggests that the vitellogenesis-stimulatory role is carried out by the SG-MIH. To our knowledge, the current study is the second description of the effect of MIH neuropeptide on *VtG *mRNA in the hepatopancreas of a mature vitellogenic female crustacean.

In light of the mutual antagonism of molt and reproduction, the coordinated control of these two processes in crustacean species that continue to molt after puberty may require a complex regulatory system to allow both events [1]. The control may be exerted by multiple CHH and MIH isoforms, which indeed are found in non- crab species such as penaeids, crayfish, and lobsters [15]. It also can be controlled by pleiotropic neuropeptides in species carrying only a few neuropeptide forms, such as *C. maenas *[17,18] and *Necora *(unpublished observation) that do molt during their adulthood. Low ecdysteroid circulating levels were also observed in another anecdysial crab, the *C. opillio *[42], suggesting that terminal anecdysis is associated with remarkably low ecdysteroids levels (lower than intermolt juveniles levels) in the hemolymph. Nevertheless, the two groups (terminally anecdysial or those that molt during their adulthood) may differ only in the duration of the intermolt stage, i.e., extended intermolt period in species like the female *C. sapidus*. Altogether, based on the low ecdysteroid level in the hemolymph compared to high MIH and CHH levels, and the potentially functional YO, we conclude that the activity of the YO is suppressed by MIH in the mature female *C. sapidus*. Furthermore, our results suggest that the SG of adult female *C. sapidus *does not contain another vitellogenic regulatory factor(s) (Fig. 7).

## Conclusion

The current study tested the possible regulatory roles of SG-MIH, CHH and CPRP in vitellogenesis, *in vitro*. The results show that MIH regulates vitellogenesis in a vitellogenic stage dependent manner: MIH is stimulatory at the levels of both transcription and translation of VtG at mid-vitellogenesis but its effect seems different at early stages. In light of the role of MIH in vitellogenesis, we infer that MIH may play a dual role in females of crab species that exhibit a terminal anecdysis upon sexual maturity in their life cycle: maintaining intermolt, while regulating vitellogenesis.

## Methods

### Animals

Female *C. sapidus *were captured in the Rhode River (a tributary of the Chesapeake Bay, USA) transported in aerated water, and held in recirculating 4.5 cubic meter tanks at environmental conditions matching the Bay for 2–5 days before dissection. Ovarian development was staged based on ovarian weight and gamete size according to criteria established previously [43]. Stage early 2 (E2) represents the beginning of vitellogenesis where oocyte size is small and similar to those at stage 1, but vitellin (yellow in color) starts to accumulate within the oocyte. This stage is the earliest point of detecting both *VtG *mRNA and VtG protein [3].

### Batch purification and quantification of native CHH, native MIH, and recombinant MIH

Neuropeptides of sinus glands (SG) were purified using RP-HPLC as described [44]. Amino acid analyses were carried out for the quantification of the purified native MIH, native CHH, and recombinant MIH (rMIH) using the *o*-phthalaldehyde pre-column derivation method as described [16].

### CHH and MIH concentrations in the hemolymph

**Figure 8:**
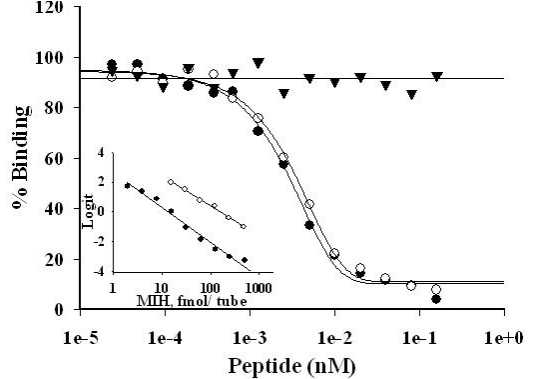
***C. sapidus *MIH antibody demonstrates high and equal specificity to MIH and rMIH and does not cross react with CHH**. Standard curves of MIH, rMIH and CHH with MIH antibody. Neuropeptides at concentrations ranging from 0.25 pM to 1 nM were applied to RIA using anti-MIH raised against rMIH produced in *Drosophila *S2 cells at 1:20,000 final dilution. Close circles- nMIH, open circles- rMIH, triangles- CHH. Inset- Parallelism of nMIH and diluted hemolymph tested by MIH RIA and shown in linear regressions. Close circles- nMIH, open circles- hemolymph.

Hemolymph samples were collected and frozen at -20°C until processed. Neuropeptides were extracted from 1 ml hemolymph by adding isopropanol to 40% (v/v) for CHH extraction followed by centrifugation at 2000 rpm for 30 min at 4°C. The resulting pellet was re-extracted with 60% isopropanol (for MIH extraction) and pelleted as before. Supernatants were pooled and dried under vacuum (SpeedVac, Jouan) before being analyzed by RIAs as described [45]. The antiserum used in MIH RIA was generated in rabbits (Proteintech Group Inc.) against recombinant MIH with a His Tag at its C terminus, produced in an S2 *Drosophila *cell expression system. Both native [^125^I] MIH and [^125^I] CHH were prepared using a chloramine-T labeling method. [^125^I] MIH or [^125^I] CHH was separated from free [^125^I] on a PD 10 column (GE Healthcare) as described [16]. Specific activities were approximately 300–500 Ci/mmol. A standard curve of MIH RIA ranging from 0.1–1000 fmol/tube was prepared with native MIH and a final dilution of MIH antiserum at 1:20,000 (Fig. 8). The sensitivity of typical MIH RIAs was between 1.2 10^-12 ^and 2.5 × 10^-11 ^M, with an ED_50 _value of 5.73 ± 0.02 × 10^-10 ^M (N = 3) and the detection limit of 1.2 × 10^-12 ^M. A serial dilution of hemolymph was run in parallel to the native MIH (Fig. 8 insert). The detailed descriptions for antiserum production and RIA of CHH were previously provided [35]. The ED_50 _value of CHH was 4.00 ± 0.02 × 10^-10 ^M (N = 3). The RIA data were analyzed and plotted using the SOFTMax PRO v1.2.0 software (Molecular Devices).

### Ecdysteroid Radioimmunoassay

Hemolymph samples (10 μl) were analyzed for the total concentration of ecdysteroid using an Ecd-RIA with ecdysone specific antiserum and [^3^H] PoA (Perkin Elmer) and Ecd antiserum, as described [18]. Ecdysone served as a standard at concentrations ranging from 2.5 ng to 30 pg/tube. The results were analyzed using the AssayZap program (Biosoft) and the EC_50 _value of Ecd-RIA was 100 ± 10 pg/tube (n = 10).

### Quantitative PCR (QPCR) analysis of *VtG *in the hepatopancreas and VtG ELISA

Total RNA was extracted using the RNeasy mini kit (Qiagen). cDNA preparation and quantitative PCR (QPCR) analysis were conducted using the procedure described for the measurement of hepatopancreas *VtG *mRNA and with normalization against arginine kinase [3]. *HnVtG *RNA was measured by QPCR analysis using a pair of primers located in intron #1 of the vitellogenin gene based on a 1539 bp partial sequence amplified from genomic DNA (GenBank accession number EU293808): Forward primer – 5'GTTCCCTGCCTGGCTTCA3'; Reverse primer – 5'CGGCTGTCGAGGTGATTATGA3'. This partial *VtG *gene served as a template for the cRNA used in the standard curve. To avoid genomic DNA amplification, total RNA was treated with 1 unit/1 μg RQ1 DNase (Promega) prior to reverse transcription, and the results obtained from cDNA amplification were corrected by subtracting the levels of non-reverse transcribed RNA amplification (no-RT control).

Vitellogenin levels in the incubation medium were determined by a competitive VtG ELISA. The media proteins were precipitated with 60% ammonium sulfate prior to the ELISA procedure as described [3]. When VtG levels were measured in the tissues and media, both were homogenized together in a "tissuelyser" (Qiagen) before ammonium sulphate precipitation.

### Incubation *in vitro *of hepatopancreas fragments with neuropeptides, actinomycin D or cycloheximide

Hepatopancreas tissue was removed from vitellogenic females and washed in 10 volumes of ice-cold Medium-199 medium (Sigma) (adjusted with NaCl to osmolarity of 960 mmol/kg), with gentle agitation on ice for 2–3 h, and 3 medium exchanges. Hepatopancreas fragments (~10 mg each) were directly weighed into 400 μl medium containing 100 μg/ml BSA, 1× protease inhibitors cocktail for tissue culture (Sigma), and with or without the tested neuropeptide in 24 well plates (Corning). The plates were incubated at 23–24°C with gentle agitation for 6 h. To test if other factors in the SG have roles in vitellogenesis, all HPLC fractions of SG extracts were pooled excluding crustacean hyperglycemic hormone precursor related peptide (CPRP), CHH, and MIH, and were also examined.

To further define the specific action of MIH, hepatopancreas fragments were exposed to actinomycin D (Sigma) or cycloheximide (Research Products international Corp.) at a final concentration of 0.5 and 10 μM in the presence of MIH. The actinomycin D results were compared to those of MIH treatment. The cycloheximide results were compared to those of MIH + 0.1% v/v EtOH treatment, since EtOH was used as a vehicle for this reagent. In these experiments, VtG levels were measured in the medium and tissue and the results were obtained as μg/mg total protein of both tissue and medium combined, before conversion into % of control. For the measurement of *hnVtG *RNA in the hepatopancreas, which indicates a *de novo *synthesis of newly transcribed and unprocessed mRNA, and reflects the actual transcription rates [46,47], incubation periods of 1 and 2 h were initially compared. Consequently, a 1 h incubation period was set for all experiments measuring *hnVtG *RNA.

In general, control treatments were tested in sextuplicate and all other treatments in quadruplicates. At the end of the experiment, the tissue fragments were frozen at -80°C until further analyses for *VtG *mRNA and *hnVtG *RNA QPCR, as described above. The results of several independent experiments were pooled, compared, and converted to % of control due to high individual variations in VtG levels.

### Statistical analysis

The data obtained from QPCR, RIA, and ELISA is presented as the mean ± SEM. The results were subjected to GraphPad Instat 3 program (GraphPad Software). The data were analyzed using one-way ANOVA followed by Tukey-Kramer multiple comparison test. In all cases, a statistical difference was accepted at P ≤ 0.05.

## Abbreviations

MIH: molt-inhibiting hormone; CHH: crustacean hyperglycemic hormone; QPCR: quantitative PCR; *VtG*: vitellogenin mRNA; ELISA: enzyme linked immunosorbent assay; RIA: radioimmunoassay; VtG: vitellogenin protein.

## Competing interests

The authors declare that they have no competing interests.

## Authors' contributions

NZ carried out the concept, experimental design, and acquisition, analyses, and interpretation of data, and drafted and revised the manuscript including tables and figures. JMT participated in the discussions and revision of the manuscript. YZ involved in the acquisition of funding and contributed to discussions. JSC was involved in the acquisition of funding, contributed to the concept, experimental design, analyses, and interpretation of data, and revised the manuscript. All authors read and approved the final manuscript.

## Supplementary Material

Additional file 1 **Ribosomal RNA stability during 6 h incubation of hepatopancreas fragments**. Gel electrophoresis of total RNA, extracted from hepatopancreas fragments before and after 6 h incubation, demonstrates that ribosomal RNA remains intact after 6 h incubation. Click here for file
