# Supplementary material for: Molt-inhibiting hormone stimulates vitellogenesis at advanced ovarian developmental stages in the female blue crab, Callinectes sapidus 1: an ovarian stage dependent involvement
Source: Saline Syst. 2009 Jul 7;5:7. doi: 10.1186/1746-1448-5-7 (PMC2715418; doi:10.1186/1746-1448-5-7)
Supplement: Additional file 1 — Ribosomal RNA stability during 6 h incubation of hepatopancreas fragments. Gel electrophoresis of total RNA, extracted from hepatopancreas fragments before and after 6 h incubation, demonstrates that ribosomal RNA remains intact after 6 h incubation. [file 1746-1448-5-7-S1.ppt]

## Slide 1
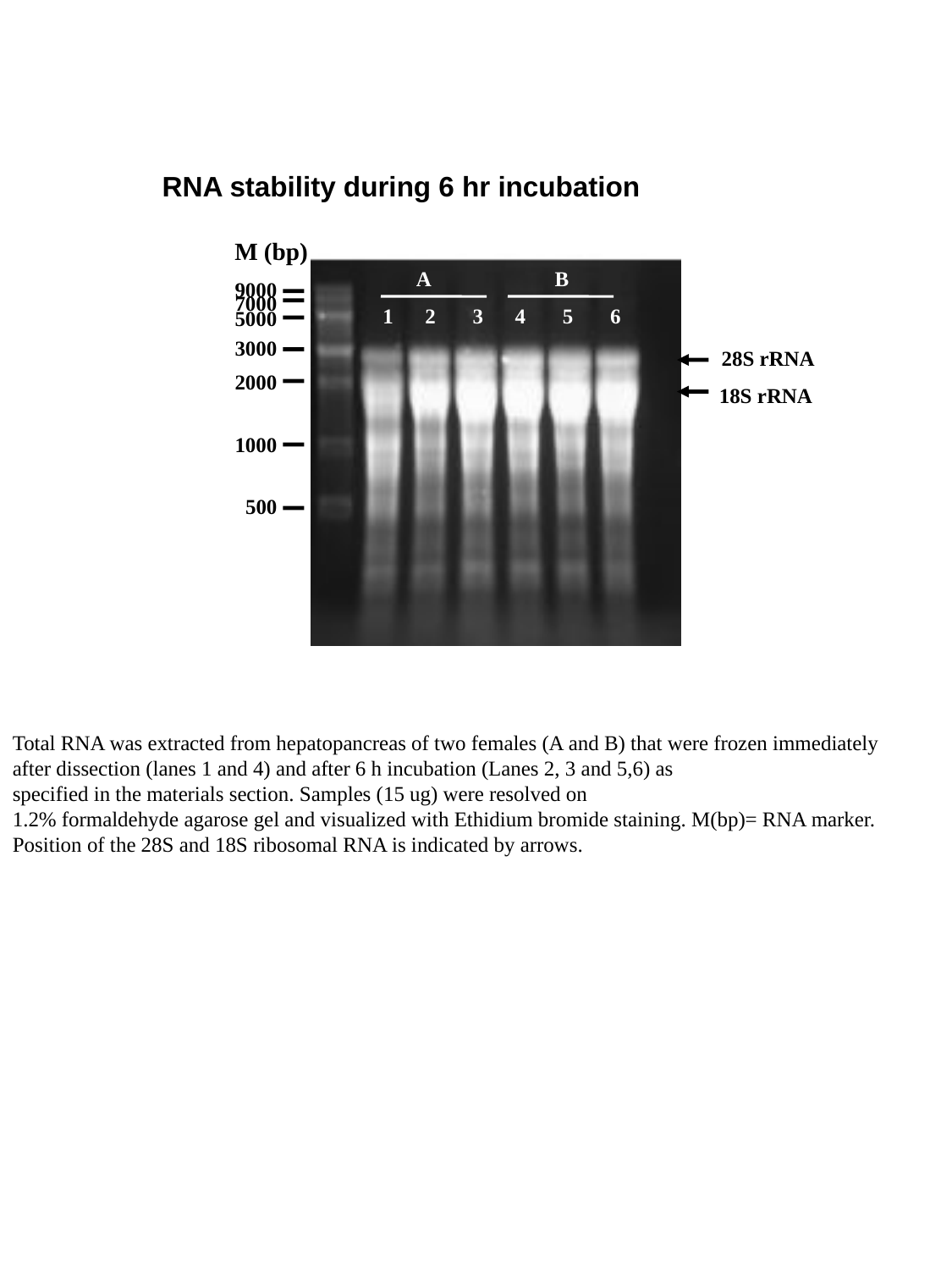

RNA stability during 6 hr incubation
M (bp)
9000
7000
5000
3000
2000
1000
500
A
B
1 2 3 4 5 6
28S rRNA
18S rRNA
Total RNA was extracted from hepatopancreas of two females (A and B) that were frozen immediately after dissection (lanes 1 and 4) and after 6 h incubation (Lanes 2, 3 and 5,6) as
specified in the materials section. Samples (15 ug) were resolved on
1.2% formaldehyde agarose gel and visualized with Ethidium bromide staining. M(bp)= RNA marker. Position of the 28S and 18S ribosomal RNA is indicated by arrows.
